# Chemical ocular trauma outbreak caused by cosmetic hair braiding and modeling ointments in Brazil

**DOI:** 10.5935/0004-2749.2024-0321

**Published:** 2025-09-10

**Authors:** Ana Cecília Carvalho Torres, Gerson Gomes da Nóbrega Filho, Analívia Barros da Costa Oliveira, Ciro Arruda Câmara Virgolino, Camila V. Ventura

**Affiliations:** 1 Department of Ophthalmology, Fundação Altino Ventura, Recife, PE, Brazil; 2 Department of Research, Fundação Altino Ventura, Recife, PE, Brazil; 3 Hospital de Olhos de Pernambuco, Recife, PE, Brazil

**Keywords:** Cosmetics, Hair preparations, Eye injuries, Burns, chemical, Eye burns, Keratitis, Cornea, Corneal diseases, Visual low

## Abstract

**Purpose:**

To report the ophthalmological signs, symptoms, and clinical management observed during an unprecedented outbreak of chemical ocular injuries related to cosmetic hair ointments in Brazil.

**Methods:**

This descriptive, cross-sectional study reviewed medical records of patients treated at the emergency center of *Fundação Altino Ventura* for chemical ocular trauma associated with cosmetic hair ointment use between February 2022 and February 2023. Records with incomplete medical information were excluded.

**Results:**

The study included 168 patients (95.2% [n=160] female), with a mean age of 30.8 ± 9.7 years. The most frequently reported symptoms at presentation were pain (167/168, 99.4%) and photophobia (92/168, 54.8%). Severe pain was reported by 137 patients (80%). Keratitis was present in 280 of 336 eyes (83.3%), conjunctival hyperemia in 256 eyes (76.4%), and corneal abrasions in 174 eyes (51.8%). A decrease in visual acuity (worse than 20/25) was documented in 18.5% (31/168) of cases. Lubricants, antibiotics, and re-epithelialization ointments were prescribed to 64.8% (109/168) of the patients. Topical corticosteroids and oral vitamin C were administered to 34% (57/168) and 1.2% (2/168) of patients, respectively. Followup visits were required in 19% (33/168) of cases.

**Conclusion:**

The outbreak of chemical ocular injuries linked to cosmetic ointments used for braiding and hair modeling in Brazil was marked by intense ocular pain, conjunctival hyperemia, keratitis, and corneal abrasions. Most patients were treated with lubricants, antibiotics, and re-epithelialization ointments, although approximately one-fifth required followup care, and one-third received additional treatment with either topical corticosteroids and/or oral vitamin C.

## INTRODUCTION

Ocular trauma is a significant cause of visual impairment, particularly in developing countries, with an estimated 90% of cases being preventable^(1)^. Chemical injuries account for approximately 7%–10% of all eye trauma incidents^(1)^. These injuries often affect both eyes, with the cornea being the most severely impacted ocular structure^(2)^.

The cornea is densely innervated, and its integrity is vital for maintaining both the functionality of the ocular surface and its refractive characteristics^(2)^. However, exposure to chemical agents can result in complications such as keratitis, corneal abrasions, and ulcers, potentially leading to vision loss^(3)^. Preventative measures, timely diagnosis, and appropriate management are essential to reduce the risk of permanent damage and the necessity for surgical treatment^(3)^.

In early 2022, Brazil experienced an outbreak of chemical ocular injuries linked to cosmetic ointments used for hair styling and braiding, which coincided with the Carnival period^(4)^. Initial data showed that more than 250 individuals sought emergency ophthalmologic care within just one week^(4)^. Among the symptoms reported, temporary vision loss raised significant concern and was linked to direct contact of the eyes with these products^(5)^. Since these ointments are typically left in the hair for prolonged periods, exposure to water—such as from rain, swimming pools, or showers—can cause the substance to run down the face and enter the eyes, resulting in chemical damage^(6)^.

In response, the Brazilian National Health Surveillance Agency (ANVISA) issued a resolution in March 2022 banning the manufacturing, distribution, and use of Omegafix Braiding Ointment^(7)^. Despite this measure, cases of chemical ocular trauma continued to increase, leading to the market withdrawal of nearly 3,000 hair braiding and styling ointments by December 2023^(8)^.

Given the epidemic characteristics of these occurrences, the present study aimed to detail the ophthalmologic signs and symptoms, as well as the clinical management strategies, observed during the unprecedented outbreak of chemical ocular injuries related to cosmetic hair ointments in Brazil from February 2022 to February 2023.

## METHODS

This descriptive, cross-sectional study reviewed the medical records of patients who presented with ocular complaints after the use of cosmetic hair modeling ointments at the Ophthalmology Emergency Center of *Fundação Altino Ventura* (FAV) in Recife, Brazil, between February 2022 and February 2023. This study received approval from the institutional ethics committee of FAV (protocol number 5.866.610) and was conducted in accordance with the principles outlined in the Declaration of Helsinki.

Patients whose medical records were incomplete were excluded from the analysis. Quantitative data were reported as means and standard deviations, while qualitative data were presented as absolute and relative frequencies. Statistical analyses were performed using jamovi project software, version 2.3.28.

## RESULTS

A total of 168 patients (336 eyes) were included in the study, of whom 160 (95.2%) were female and 82 (48.8%) resided in Recife, Brazil. The mean age at the time of admission was 30.8 ± 9.7 years (Table 1). The most frequent reported ocular symptoms were pain (167/168, 99.4%) and photophobia (92/168, 54.8%) (Table 2). According to the Visual Analog Scale for pain assessment, 137 out of 168 patients (80%) rated their pain at admission as severe (scores between 7 and 10 out of 10), while 33 patients (20%) rated it as moderate (scores between 5 and 6 out of 10).

**Table 1:** Demographic characteristics of patients (n=168)

Variables	Values
Age (years) (*mean ± standard deviation)*	30.8 ± 9.7
Sex (n%)	
Female	160 (95.2)
Male	8 (4.8)
Origin (n%)	
Recife	82 (48.8)
Olinda	26 (15.5)
Other regions	60 (35.7)
Followup (days) (n=36) Mean (min–max)	3.5 (1–10)

**Table 2:** Primary complaints upon initial emergency room presentation (n=168) and biomicroscopy findings (n=336 eyes)

Variables	n (%)
Main complaints (n=168 patients)	
Pain	167 (99.4)
Photophobia	92 (54.8)
Tearing	55 (32.7)
Blurry vision	31 (18.5)
Foreign body sensation	27 (16.1)
Periorbital edema	10 (6)
Conjuntival discharge	5 (3)
Biomicroscopy (n=336 eyes)	
Keratitis	280 (83.3)
Conjunctival hyperemia	256 (76.4)
Corneal abrasion	174 (51.8)
Anterior chamber cells	44 (13.1)
Conjunctival discharge	34 (10.1)
Ciliary injection	6 (1.8)

**Figure 1:**
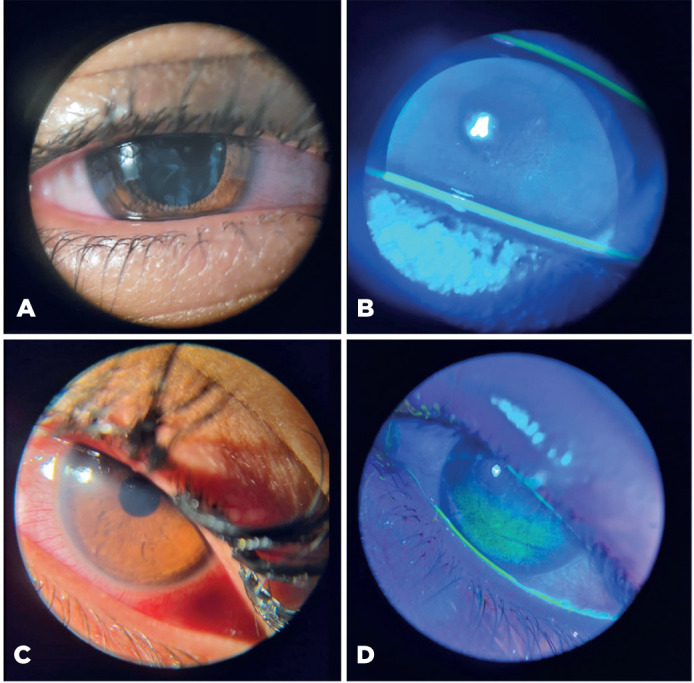
Various levels of ocular injury caused by chemical trauma from styling hair pomades. (A) Mild conjunctival hyperemia following exposure to a styling hair pomade. (B) Mild keratitis shown by fluorescein staining under blue light. (C) Moderate conjunctival hyperemia with ocular inflammation after exposure to a styling hair pomade. (D) Moderate keratitis with fluorescein uptake visible under blue light.

**Figure 2:**
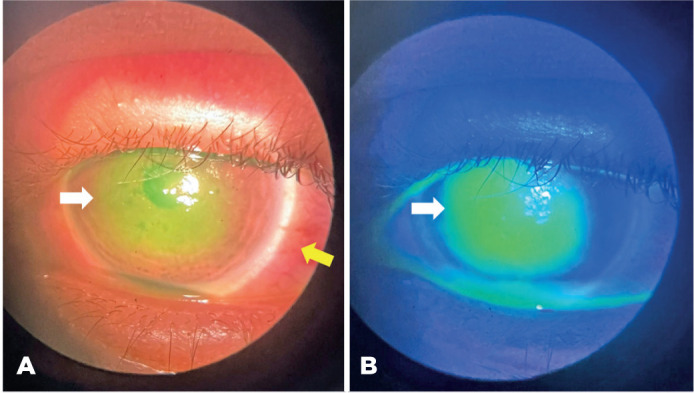
Severe ocular injury related to exposure to a styling hair pomade. (A) Severe conjunctival hyperemia with a clearly defined limbus (yellow arrow) and widespread central keratitis (white arrow). (B) Severe keratitis demonstrated under blue light with intense fluorescein uptake (white arrow).

Biomicroscopy during the initial assessment revealed keratitis in 280 of 336 eyes (83.3%), conjunctival hyperemia in 256 eyes (76.4%), and corneal abrasion in 174 eyes (51.8%) (Figures 1 and 2). The presence of anterior chamber cells was documented in 44 eyes (13.1%), and ciliary injection was observed in six eyes (1.8%) (Table 2). All patients (168/168, 100%) received treatment. The majority (109/168, 64.8%) were prescribed a combination of lubricants, antibiotics, and re-epithelialization ointments. In addition to these, topical corticosteroids were prescribed for 57 patients (34%). In the most severe cases (2/168, 1.2%), treatment also included oral doxycycline and oral vitamin C (Table 3).

**Table 3:** Treatments administered (n=168 patients)

Treatment prescribed	Drugs	n (%)
1	*Lubricant* *Topical antibiotics* *Re-epithelialization* *ointment*	109 (64.8)
2	*Lubricant* *Topical antibiotics* *Re-epithelialization* *ointment* *Corticosteroids*	57 (34)
3	*Lubricant* *Topical antibiotics* *Re-epithelialization* *ointment* *Corticosteroids* *Oral doxycycline* *Oral vitamin C*	2 (1.2)

No cases exhibited visual sequelae. Thirty-two patients (19%) required one followup visit, and one patient (0.6%) returned for two followup visits, with a mean followup interval of 3.5 ± 1.7 days (range, 1–10 days). The specific brand and composition of the ointment used were identified in ten cases (6%). The most frequently detected ingredients were Ceteareth-20 (nine out of ten cases, 90%), ethylenediaminetetraacetic acid (EDTA) (8/10, 80%), and propylene glycol (6/10, 60%) (Table 4).

**Table 4:** Components of the ointments

Chemical components of the hair modeling ointments	n (%)
*Ceteareth-20*	9 (90)
*Dissodium EDTA*	8 (80)
*Propylene glycol*	6 (60)
*Methylchloroisothiazolinone (MCI)*	4 (40)
*Methylisothiazolinone (MI)*	3 (30)

## DISCUSSION

Chemical eye burns pose a considerable public health concern and warrant focused attention from both healthcare professionals and the general population^(9)^. These injuries are considered ophthalmic emergencies and account for approximately 11.5%–22.1% of eye trauma cases globally^(9)^. The primary agents responsible for such burns are acids and alkalis. Notably, substances such as caustic soda, ammonia, potassium hydroxide, and calcium hydroxide are associated with the greatest frequency and severity of injuries. These chemicals can inflict serious, and in some cases irreversible, ocular damage, potentially resulting in permanent vision loss^(10^,^11)^.

Toward the end of 2022, a surge in chemical eye burns related to the use of hair styling ointments was reported during Brazil’s Carnival season. This phenomenon drew national attention and became known as “the hair braiding ointments outbreak”^(5^,^8)^. As a precaution, in March 2023, the Brazilian National Health Surveillance Agency (ANVISA) issued a temporary ban on all hair styling ointments pending further investigation^(12)^.After examining the formulations of the ointments, it was found that most products causing severe adverse eye effects contained Ceteareth-20, which is commonly used in cosmetics as an emulsifying agent and belongs to the ethoxylated fatty alcohol family. Following this analysis, the regulatory agency imposed a precautionary ban on products containing more than 20% Ceteareth-20, allowing some of the recalled products to return to the market^(10)^. However, in one case (1 out of 10, 10%) in this study, Ceteareth-20 was not detected in the hair styling ointment, suggesting that other substances might also contribute to the adverse effects.

The second most frequent compound identified in the ointments was EDTA, used in cosmetics to enhance the efficacy of preservatives^(13^,^14)^. At certain concentrations, EDTA can cause serious eye damage, including irreversible blindness^(13^,^14)^. The third most commonly found ingredient was propylene glycol, an alcohol used as a preservative in cosmetic products, which can also be toxic to the eyes at inappropriate concentrations^(13)^. Methylchloroisothiazolinone (MCI) and methylisothiazolinone (MI), parabens used as preservatives, were also detected and are known to be toxic when presented in excessive amounts^(15)^.

In this study, a high incidence of severe pain was observed, followed by photophobia. These symptoms can be attributed to the extensive damage chemical eye burns cause to the ocular surface epithelium, including the cornea, conjunctiva, and limbal stem cells, which results in pain and may lead to temporary or permanent vision loss^(16)^. Furthermore, the severity of ocular damage caused by chemical agents depends on the concentration, pH of the substance, and the duration of exposure^(17)^. The chemical ingredients in the braiding hair ointments, when coming into contact with water (such as from showers, rain, or swimming) or sweat, increase the risk of exposure to the ocular surface^(17)^. This study demonstrated that the use of these products may result in keratitis, conjunctival hyperemia, and corneal abrasions.

Previous research indicates that most chemical eye injuries affect young males, tend to be bilateral, and generally have a favorable prognosis^(17^-^19)^. Interestingly, in the present study, most affected patients were young females with bilateral involvement. The predominance of females likely reflects the fact that these products are predominantly used for hair braiding. Moreover, while studies report that accidental ocular burns typically occur at home or in the workplace^(18)^, our study found that cases occurred in a recreational setting, during Carnival festivities, where these hair products are commonly used for styling or braiding.

Fortunately, all cases in this study resolved with appropriate treatment. Previous studies indicate that early intervention in chemical ocular burns leads to a better prognosis^(18^-^20)^. Standard therapy involves using agents that promote epithelial healing, reduce inflammation, and prevent scarring complications^(18^-^22)^. In our study, treatments varied but mainly focused on lubricants, antibiotics, and re-epithelialization of ointments. It is important to note that nearly 20% of patients required at least one followup visit, and one-third received additional treatment with oral vitamin C and/or topical steroids, suggesting limbal involvement in more severe cases.

The main limitations of this study include the lack of information about the specific time elapsed between chemical exposure and emergency room admission, as well as the concentration of the chemical agents involved. Nevertheless, we aimed to characterize the affected patients during the hair ointment outbreak in Brazil, focusing on their primary complaints, ocular findings at admission, and management to better define the sample. Given these limitations, further studies are needed to improve understanding of the damage caused by these chemical agents on the ocular surface and the concentrations at which they are harmful. Moreover, while sales data for braiding products could help estimate the incidence of related injuries, such information was not available during this research. However, it is worth noting that these products are widely sold through various outlets, including pharmacies, supermarkets, specialized cosmetic stores, and online platforms.

In conclusion, this study reports the clinical signs and treatment of chemical ocular trauma linked to the use of cosmetic hair braiding and styling ointments in Brazil. The majority of patients experienced severe eye pain, keratitis, conjunctival hyperemia, and corneal abrasions that required topical therapy. Although the symptoms were acute, all cases improved with treatment, and no permanent visual damage was detected. These results highlight the need for further investigation into the chemical ingredients involved and their ocular toxicity, as well as the development of preventive measures. Future research addressing the long-term consequences of these injuries and potential regulatory measures may help prevent similar outbreaks.

## Data Availability

The datasets generated and/or analyzed during the current study are included in the manuscript.
